# Exploring health literacy categories among an Iranian adult sample: a latent class analysis

**DOI:** 10.1038/s41598-023-49850-3

**Published:** 2024-01-08

**Authors:** Mir Saeed Yekaninejad, Ahmadreza Hajiheidari, Mehran Alijanzadeh, Rafat Yahaghi, Zahra Karimi, Jalal Rahmani, Nahid Yazdi, Elahe Jafari, Hashem Alijani, Narges Zamani, Razie Fotuhi, Elham Taherkhani, Zeinab Buchali, Masoume Zarenejad, Narges Mahmoudi, Najmeh Shahmahdi, Leila Poorzolfaghar, Safie Ahmadizade, Azam Shahbazkhania, Marc N. Potenza, Chung-Ying Lin, Amir H. Pakpour

**Affiliations:** 1 Department of Epidemiology and Biostatistics, School of Public Health, Tehran University of Medical Sciences, Poursina St., Keshavarz Blvd., Tehran, Iran; 2 Independent researcher/Freelancer, Tehran, Iran; 3 Social Determinants of Health Research Center, Research Institute for Prevention of Non-Communicable Diseases, Qazvin University of Medical Sciences, Qazvin, 3419759811 Iran; 4 Departments of Psychiatry and Neuroscience and the Child Study Center, Yale University, New Haven, USA; 5 Connecticut Council on Problem Gambling, Hartford, USA; 6 Connecticut Mental Health Center, New Haven, USA; 7 Wu Tsai Institute, Yale University, New Haven, USA; 8 Institute of Allied Health Sciences, College of Medicine, National Cheng Kung University, Tainan, 701401 Taiwan; 9 Biostatistics Consulting Center, National Cheng Kung University Hospital, College of Medicine, National Cheng Kung University, Tainan, 701401, Taiwan; 10 Department of Public Health, National Cheng Kung University, Tainan, 701401 Taiwan; 11 Department of Occupational Therapy, College of Medicine, National Cheng Kung University, Tainan, 701401 Taiwan; 12 Department of Nursing, School of Health and Welfare, Jönköping University, Gjuterigatan 5, 55318 Jönköping, Sweden

**Keywords:** Psychology, Health care

## Abstract

General and electronic health literacy are important factors engaging in healthy behaviors and maintaining good health. The present study explored demographic factors associated with general and electronic health literacy in the Iranian adult population. Via stratified cluster sampling, trained interviewers visited adult residents in Qazvin Province, Iran between January, and April 2022. The participants (N = 9775; mean age = 36.44 years; 6576 [67.3%] females) completed the Health Literacy Instrument for Adults (HELIA) assessing health literacy and the eHealth Literacy Scale (eHEALS) assessing electronic health literacy. Demographic data, including age, gender, educational level, marital status, and living location (city or rural), were collected. Latent class analysis (LCA) was used to classify the participants into different health literacy/electronic health literacy levels. The relationships between health literacy/electronic health literacy levels and demographic factors were examined using χ^2^ or analysis of variance. The LCA used HELIA scores to suggest five classes of health literacy and eHEALS scores to suggest three classes of electronic health literacy. For general and electronic health literacy, similar relationships were with demographic factors: females as compared with males had better general/electronic health literacy; younger people as compared with older people had better general/electronic health literacy; higher educational level was associated with better general/electronic health literacy; and city residents as compared with rural residents had better general/electronic health literacy. In conclusion, Iranian governmental agencies may wish to target on males, older adults, people with low educational level, and rural residents to improve their health literacy.

## Introduction

The importance of health literacy has been well documented. Health literacy can promote engagement in healthy behaviors and reduce engagement in unhealthy ones^1,2^. When people engage in healthy behaviors (e.g., exercising) and reduce unhealthy one (e.g., smoking), their health, including life expectancy and life quality, are often substantially improved^3–6^. Such effects of health literacy may relate to specific qualities: health literacy may involve people’s abilities in reading, writing, comprehending, and understanding useful health information^7^. Moreover, the World Health Organization (WHO) indicated that high levels of health literacy will motivate people to promote and maintain good health^8^.

Given technological advances, health literacy has evolved into digital forms (i.e., electronic health literacy)^9,10^. Accordingly, health literacy includes general and electronic health literacy. Electronic health literacy has then been defined as “the ability to gather and appropriately process health information retrieved online”^11^. The importance of electronic health literacy is growing due to continuously advancing technologies. People with higher levels of electronic health literacy have demonstrated better knowledge of colorectal cancer and more engagement in cancer screening^12^. Electronic health literacy is an important factor associated with better preventive behaviors (e.g., medication adherence) among patients with heart failure and results in better health outcomes^13^. In addition, electronic health literacy was found to be associated with better mental health among adult population^14^, with better behavioral and cognitive performance among older people^15^, and higher adherence to health-promotion behaviors among adolescents^16^.

Given that health literacy and electronic health literacy are important for people to obtain and maintain healthy mental and physical and fitness, it is important to examine potential demographic factors associated with general and electronic health literacy. To date, several demographic factors have been associated with general or electronic health literacy, but findings are sometimes mixed. For example, males have demonstrated better health literacy than females^17,18^, females have demonstrated better health literacy than males^19–21^. and no significant differences in health literacy have been observed between genders^22^. Most studies have found that older people (especially those aged 65 years or above) demonstrate poorer health literacy as compared with t younger individuals^20,22–27^, with an exception being an Australian study showing that older people had slightly better health literacy than younger people^17^. Most studies showed that people having higher educational levels as compared with those having lower educational ones had better health literacy^17–21,26,28^. Moreover, people living in rural areas have demonstrated similar health literacy score to those living in urban areas^17^; single and married people have demonstrated better health literacy than those who are widowed, divorced, or separated^22^. As a result, health literacy may have different associations with gender according to the samples studied, with arguably the most consistent findings with age and educational level.

Multiple factors may influence relationships between health literacy and specific demographic features. For example, cultural factors may exert differential effects on specific relationships, e.g., how older versus younger adults or males versus females are enculturated in specific societies. Additionally, the use of digital technologies maty differ across jurisdictions. Most research has been conducted in WEIRD (Western, educated, industrialized, rich and democratic) countries. As such, there is a need to study relationships between health literacy and demographic measures in non-WEIRD countries like Iran.

To the best of the present authors’ knowledge, studies assessing associations between general and electronic health literacy and demographic factors have observed raw scores of general or electronic health literacy. Although using the raw scores can help understand associations, they may not help identify if a person is classified in a certain level of general or electronic health literacy, such as a high, middle, or low level of literacy. In other words, raw scores may help identify relative levels of general or electronic health literacy (e.g., score 20 is higher than score 18) but may not distinguish group levels (e.g., if score 20 is of a high, middle, or low level). Consequently, one may not identify important demographic factors associated with degrees of general or electronic health literacy using the scores. To overcome this concern, latent class analysis (LCA) may classify people into different groups based on general or electronic health literacy. In other words, the LCA helps researchers to cluster the people into different classes (or levels) using the latent features (e.g., the health literacy and electronic health literacy in the present study) of these participants. Specifically, LCA applies fit statistics to help researchers identify appropriate latent groups to ensure the robustness of classifications^29^. LCA utilizes probability calculated from raw scores to identify the classes to which individuals belong^30^. Therefore, estimations of an individual belonging to a specific latent class is arguably less biased as compared with other clustering statistics^31^.

To better understand associations between general/electronic health literacy and demographics, we sought to classify individuals based on general/electronic health literacy levels using LCA. Consequently, levels of general/electronic health literacy classified by LCA could be used to investigate relationships with demographic features. The present study used a large sample from the general Iranian population to classify individuals based on levels of general and electronic health literacy. Next, the study assessed which demographic factors related to the LCA-identified groups.

## Methods

### Participants and data collection

This cross-sectional study was conducted on a sample from the general population of adults in Qazvin, Iran between January, and April 2022. The Ethics Committee of Qazvin University of Medical Sciences (IR.QUMS.REC.1400.225) approved the study. All methods were performed in accordance with the relevant guidelines and regulations. Using a stratified cluster sampling technique from 70 strata in the Qazvin province, paper-and-pencil questionnaires were distributed by trained interviewers to adult residents in Qazvin. Specifically, the trained interviewers contacted the adults who were selected for participation to explain the research purpose and information. After the participants agreed to participate, they were asked to come to the health centers to complete the questionnaires using paper and pen under the supervision of the trained interviewers. Moreover, we have invited 14,100 participants to participate and 9775 completed the questionnaires with a response rate of 69%. The sample size was sufficient for the LCA used in the present study given that prior methodological evidence shows that a sample size > 500 is needed for LCA^32,33^. All participants provided written informed consent before beginning the survey^34^. The detailed information regarding the study procedure has been described elsewhere^14,34,35^. Although the data of the present study are the same as those used in other research^14^, different research questions have been investigated between the present study and prior research^14^.

### Measures

#### Health Literacy Instrument for Adults (HELIA)

The HELIA is a subjective measure assessing health literacy using 33 items rated on a five-point Likert scale (1 = never; 5 = always). The 33 items were distributed in five types of health literacy: reading (four items; example item: “*Reading educational materials about health (booklets, pamphlets, leaflets) is easy for me*”), access to information (six items; example item: “*I can find health information about healthy eating*”), understanding (seven items; example item: “*I can understand the recommendations for a healthy diet*”), appraisal (four items; example item: “*I can communicate trusted health information to others*”), and decision-making/behavioral intention (12 items; example item: “*I am helath-conscious in any situation*”). Overall health literacy can be assessed using the HELIA total score (i.e., summing the 33 item scores), and higher scores indicate better health literacy^34^. The HELIA has been validated among Iranian adults with promising psychometric properties^36^.

#### eHealth Literacy Scale (eHEALS)

The eHEALS is a subjective measure assessing electronic health literacy using eight items rated on a five-point Likert scale (1 = strongly disagree; 5 = strongly agree). The eight items reflect the construct of electronic health literacy (example item: “*I know how to find helpful health resources on the Internet*”)^10^. Overall electronic health literacy can be assessed using the eHEALS total score (i.e., summing the eight item scores), and higher scores reflect better electronic health literacy^13^. The eHEALS has been validated among Iranian adults with promising psychometric properties^37^.

#### Demographic measures

Participants were asked to report their demographic information, including age, gender, educational level, living location, and marital status.

### Data analysis

LCA is a modeling technique used for identifying subgroups of individuals with unobserved but distinct patterns of responses to a set of observed categorical indicators. The LCA model was fitted based on the score of each item in both HELIA and eHEALS questionnaires. First, each person's scores were converted to 1 and 2. In this way, scores higher than three were considered as two and scores three or lower were considered as one. The LCA model was fitted using the obtained variables. The LCA model was performed in R software using the PoLCA package. Based on the literature, a conservative and reliable group of fit indices and criteria was selected to evaluate the goodness of fit of the possible LCA models: Akaike Information Criterion (AIC), Bayesian Information Criterion (BIC), the adjusted BIC (aBIC), G2, χ^2^, Entropy, Lo-Mendell-Rubin adjusted likelihood ratio test (LMRT) and Bootstrapped likelihood ratio test (BLRT). The best-fitting models are those with the best balance between smaller values on the AIC, BIC, aBIC and χ^2^ statistics, higher log likelihoods, and fewer numbers of parameters in the model. Finally, χ^2^ tests were used to examine if the participants at different levels of general or electronic health literacy differed in their demographic characteristics.

## Results

### Descriptive characteristics

A total of 9775 adults completed the study measures. The mean age of the participants was 36.44 ± 11.97 years. Most participants were female (n = 6576, 67.3%), lived in city areas (n = 7287, 74.6%), had completed university degrees (n = 3851, 39.4%), and were married (n = 6987, 71.5%).

### LCA Models for HELIA and eHEALS

**Figure 1:**
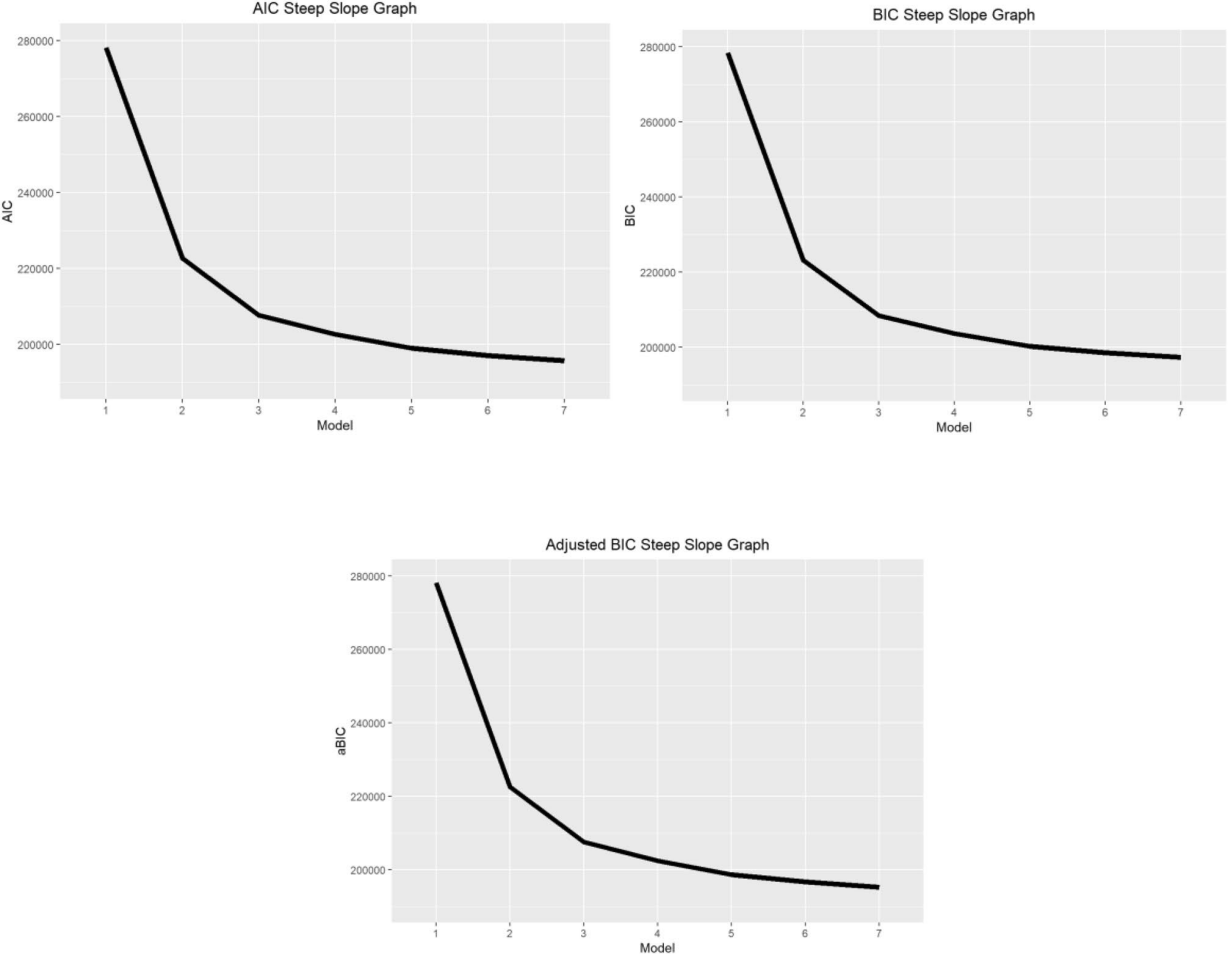
Comparison of AIC, BIC, and Adj. BIC on Steep Slope Graphs for HELIA.

**Figure 2:**
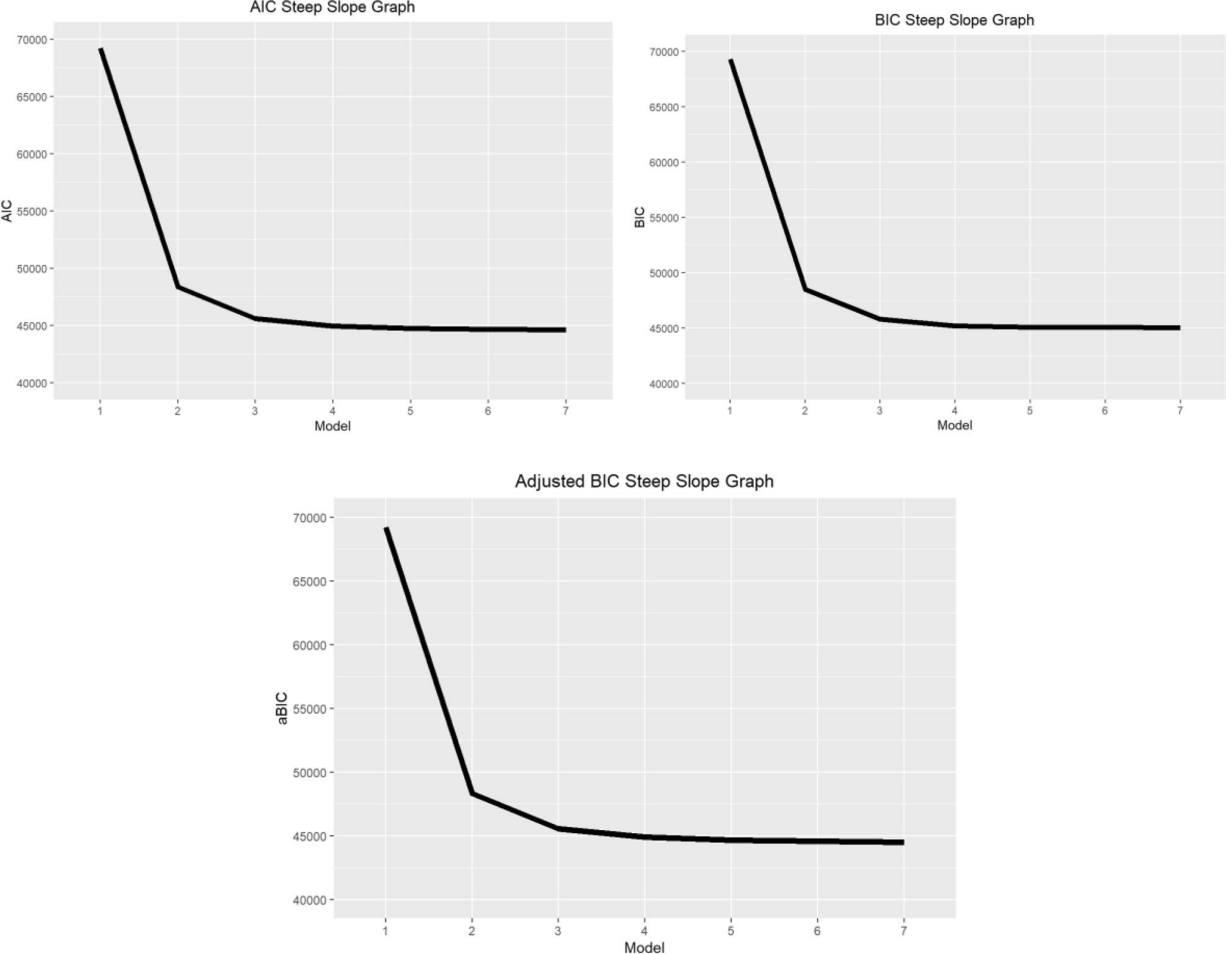
Comparison of AIC, BIC, and Adj. BIC on Steep Slope Graphs for eHEALS.

LCA resulted in a final model with five latent classes in HELIA and three latent class in eHEALS questionnaires (Table 1). Models from one-class to seven-class solutions were analyzed and compared to decide upon the number of classes with the consideration of balancing better model fit (higher log likelihood) with parsimony (fewer parameters in the model). The fit indices in each model are shown in Table 1. With increases of classes, the following criteria decreased: AIC, BIC, aBIC and χ^2^. However, only from the fourth to the fifth class were there significant decreases according to χ^2^ tests. Both the BLRD and LMRT had statistical significance (*P* < 0.001). As a result, five latent classes for HELIA were selected. Similar considerations led to three latent classes for eHEALS being selected. The slopes of the AIC, BIC, and aBIC measures are presented in Fig. 1 (for HELIA) and Fig. 2 (for eHEALS).

**Table 1:** Model fit indices of different latent class models based on scores from the Health Literacy Instrument for Adults (HELIA) and eHealth Literacy Scale (eHEALS).

No of clusters	AIC	BIC	aBIC	X2	BLRT	LMRT	Entropy	Conditional probability
HELIA								
1 cluster	278,138.4	278,366.2	278,078.1	5.896207e + 19	< 0.001	< 0.001		1
2 clusters	222,620.1	223,082.6	222,491.8	6.384271e + 10	< 0.001	< 0.001	0.939	0.672/0.328
3 clusters	207,674.8	208,372.1	207,478.6	8.714285e + 10	< 0.001	< 0.001	0.916	0.121/0.48/0.399
4 clusters	202,613.9	203,545.8	202,349.6	8.438307e + 10	< 0.001	< 0.001	0.887	0.417/0.208/0.265/0.11
5 clusters	198,995.7	200,162.4	198,663.5	2.754168e + 10	< 0.001	< 0.001	0.879	0.358/0.169/0.205/0.181/0.087
6 clusters	197,022.6	198,423.9	196,622.3	2.520531e + 10	< 0.001	< 0.001	0.867	0.148/0.18/0.077/0.338/0.078/0.178
7 clusters	195,621.1	197,257.2	195,152.9	2.000736e + 10	< 0.001	< 0.001	0.854	0.105/0.153/0.163/0.138/0.302/0.079/0.061
eHEALS								
1 cluster	69,225.78	69,281.38	69,215.55	3,961,674.0842	< 0.001	< 0.001		1
2 clusters	48,353.38	48,471.53	48,325.16	6748.9356	< 0.001	< 0.001	0.915	0.737/0.263
3 clusters	45,603.24	45,783.93	45,557.01	1930.7989	< 0.001	< 0.001	0.844	0.25/0.143/0.607
4 clusters	44,954.95	45,198.19	44,890.72	786.8742	< 0.001	< 0.001	0.816	0.141/0.126/0.614/0.12
5 clusters	44,757.39	45,063.18	44,675.16	547.3693	< 0.001	< 0.001	0.764	0.132/0.573/0.123/0.052/0.119
6 clusters	44,675.51	45,043.84	44,575.28	427.660	< 0.001	< 0.001	0.771	0.043/0.047/0.131/0.098/0.100/0.58
7 clusters	44,599.54	45,030.43	44,481.32	310.5297	< 0.001	< 0.001	0.759	0.084/0.099/0.124/0.055/0.569/0.035/0.034

*AIC* Akaike Information Criterion; *BIC* Bayesian Information Criterion; *aBIC*adjusted BIC; *LMRT* Lo-Mendell-Rubin adjusted likelihood ratio test; *BLRT* Bootstrapped likelihood ratio test.

### Class descriptions

**Figure 3:**
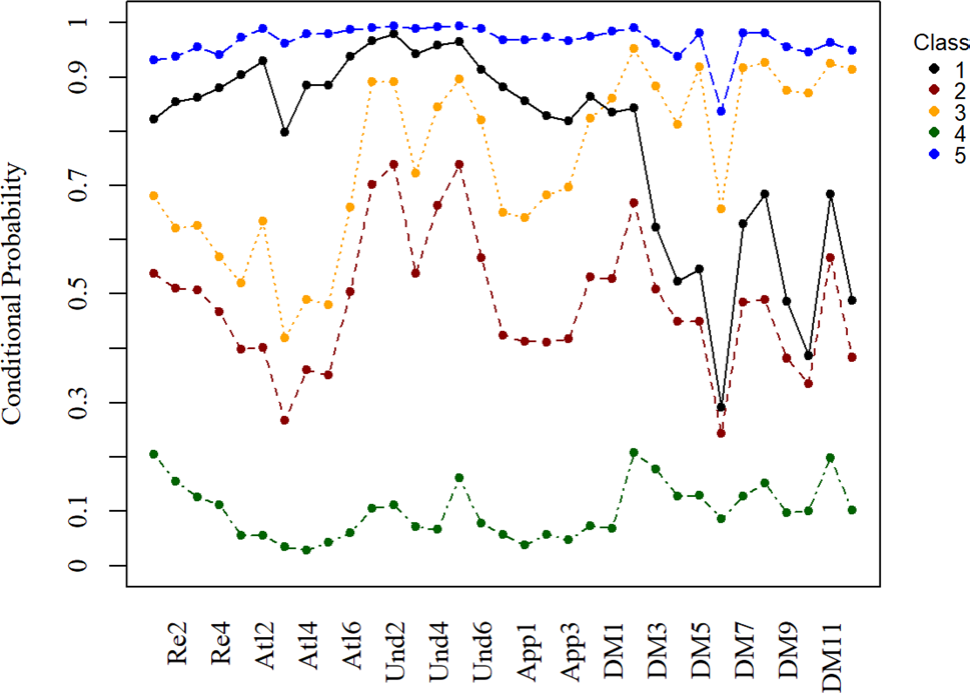
Conditional probability distribution on each item of the HELIA.

Regarding the HELIA classes, Class 1 (35.8% of the sample) was labelled as a “very high health literacy” group and was characterized by high probability of endorsement of nearly all HELIA items. Class 2 (16.9%) was labelled as a “moderate health literacy” group and characterized by strong endorsement of understanding and appraisal. Class 3 (20.5%) was labelled as a “high health literacy” group characterized by high probability of endorsement of reading, access to information, understanding and appraisal. Class 4 (18.1%) was labelled as a “low health literacy” group characterized by similar patterns with Class 2 but with lower endorsement rates. Class 5 (8.7%) was labelled as a “very low health literacy” group characterized by low endorsement of all HELIA items (Fig. 3).

**Figure 4:**
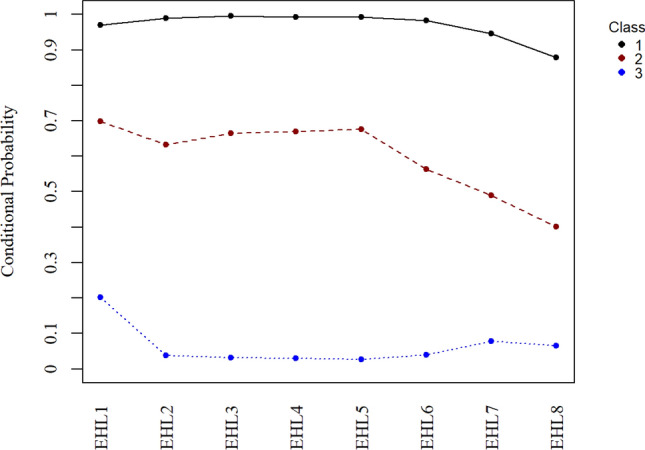
Conditional probability distribution on each item of the eHEALS.

Regarding the eHEALS classes, Class 1 (25% of the sample) was labelled as a “moderate electronic health literacy” group and was characterized by strong endorsement of how to find helpful health resources on the internet (eHEALS1), how to use the internet to answer health questions (eHEALS2), what health resources are available on the internet (eHEALS3), where to find helpful health resources on the internet (eHEALS4), and how to use the health information (eHEALS5) (Fig. 4). Class 2 (14.3%) was labelled as a “low electronic health literacy” group and was characterized by low endorsement of all eHEALS items (Fig. 3). Class 3 (60.7% of sample) was labelled as “high electronic health literacy group” and was characterized by high probability of endorsement of nearly all eHEALS items.

### Demographic characteristics relating to specific classes

Univariate analyses of sociodemographic characteristics and latent classes for both HELIA and eHEALS latent classes are shown in Supplementary Tables S1, S2, S3 and S4. All latent classes for both HELIA and eHEALS differed across gender, age, HELIA total score, eHEALS total score, education level, and living location status. Latent classes of HELIA differed across marital status but those of eHEALS did not. There were more females than males in classes reflecting better general/electronic health literacy; more younger people than older people were in classes reflecting better general/electronic health literacy; more people with higher educational levels than those with lower educational levels were in classes reflecting better general/electronic health literacy; and more city residents than rural residents were in classes reflecting better general/electronic health literacy.

## Discussion

To the best of the present authors’ knowledge, this is the first large-scale study assessing both general and electronic health literacy among a sample from the Iranian adult population analyzed using LCA. The findings indicate that individuals could be grouped into five different levels according to general health literacy and three different levels according to electronic health literacy. Higher levels of general and electronic health literacy were found in latent classes with more females, younger people, education, and city residents. Therefore, classes based on measures of general and electronic health literacy displayed largely similar relationships with multiple demographic factors. These findings suggests that electronic health literacy shares similar feature to general health literacy; that is, whether an individual could understand and use health information^7,11^.

A difference between general and electronic health literacy may involve the platform of obtaining health information (internet for electronic health literacy and no specific platform for general health literacy).

The findings that females had better health literacy than males in the present study are consistent with some prior findings^19–21^. However, other studies reported that males as compared with females had better health literacy^17,18^, whereas some found no differences between males and females^22^. The differences could reflect jurisdictional differences. For example, studied an Australian sample^17^ and studied a Turkish sample^18^. Prior studies of Iranian samples suggest that female Iranians had better general/electronic health literacy than male Iranians^19,21^, except for a study^22^. Finding no significant difference. A potential reason for the higher levels of general/electronic health literacy in women than in men could be explained by the gender roles in Iran. Specifically, Iranian women were not expected to work but to take care of the family’s health^19^. In this regard, Iranian women have time and motivations to learn health information, which subsequently reflect on their levels of health literacy. However, prior Iranian studies did not use LCA to examine relationships between health literacy and gender. The present findings are consistent with prior reports of Iranian females having better health literacy, including electronic health literacy, than Iranian males.

In agreement with other prior findings^20,22–27^, the present study found that younger Iranians had better general and electronic health literacy than older Iranians. A main reason may involve educational levels. Specifically, younger people had higher levels of education than older people, and higher levels of education were associated with better general/electronic health literacy, as evidenced by the present findings and prior research^17–21,26,28^.

Beauchamp et al. (2015) found that people living in rural areas had similar health literacy scores to those living in urban areas; however, we found that city residents in the present study had better general/electronic health literacy than rural residents. One potential explanation may involve educational levels between urban and rural regions across countries^17^. Beauchamp et al. (2015)^17^ conducted their study in Australia, which has a well-established educational system to ensure that education quality is similar between urban and rural regions^38^. In contrast, the present study was conducted in Iran, which may have more barriers in improving quality education in rural areas^39^. Potentially as a result, rural residents in the present study had poorer general and electronic health literacy than the city residents.

## Limitations and implications for practice

Study limitations warrant mention. First, although this is a large-scale study with a cluster sampling method for participant recruitment, all participants were residing in Qazvin province. Therefore, the present sample is not representative of the general Iranian population; subsequently, the present findings may not generalize to Iran or other jurisdictions. Future studies should investigate other populations. Second, both general and electronic health literacy were assessed via self-report. Although both the HELIA and eHEALS are validated instruments with promising psychometric properties in Iranian samples^34,36,37^, some participants may have misestimated their general or electronic health literacy because of factors like social desirability. Third, this is a cross-sectional design study. Therefore, causal inferences should not be drawn. Apart from the limitations and future study directions, future studies may want to use both HELIA and eHEALS to examine the associations between different forms of healthy literacy and various health outcomes. For example, a recent study using the same set of the present data found that both general and electronic health literacy were associated with better mental wellbeing via the sleep hygiene behaviors^14^. Health literacy assessed using HELIA/eHELAS may thus also be examined with other health concepts, such as physical activity and physical health.

## Conclusions

The present study found that Iranian people could be classified into five groups according to levels of general health literacy and three groups based on levels of electronic health literacy. People of female versus male gender, of younger versus older age, having higher versus lower educational levels, and living in cities (as compared with rural areas) had better general and electronic health literacy. Therefore, the Iranian government and other stakeholders focusing on improving the public health may wish to target males, older adults, people with low educational levels, and rural residents with respect to improving their health literacy.

### Supplementary Information

Supplementary Tables.

## Data Availability

All data generated or analyzed during this study are included in this published article.
