# Supplementary material for: Exploring health literacy categories among an Iranian adult sample: a latent class analysis
Source: Sci Rep. 2024 Jan 8;14:776. doi: 10.1038/s41598-023-49850-3 (PMC10774330; doi:10.1038/s41598-023-49850-3)
Supplement: Supplementary file 1 — Supplementary Tables. [file 41598_2023_49850_MOESM1_ESM.docx]

|  | | | **Groups (Mean ± SD)** |  | | | |
| --- | --- | --- | --- | --- | --- | --- | --- |
| **Variables** | Class1 | Class2 | Class3 | Class4 | Class5 | Kruskal-Wallis | p-value |
| Age, years | 35.32 ± 10.74 | 36.36 ± 11.90 | 35.20 ± 10.78 | 36.53 ± 12.67 | 40.22 ± 14.37 | 56.69 | < 0.001 |
| HELIA Score | 152.35 ± 10.15 | 132.86 ± 9.09 | 134.82 ± 9.77 | 112.64 ± 9.55 | 85.03 ± 15.59 | 5587.24 | < 0.001 |

Table S1. Univariate Analysis of the Characteristics of the Participants in Different Latent Classes based on HELIA data

Table S2. Univariate Analysis of the Characteristics of the Participants in Different Latent Classes based on eHEALS data

| **Variables** | Class1 | Class2 | Class3 | Kruskal-Wallis | p-value |
| --- | --- | --- | --- | --- | --- |
| Age, years | 36.13 ± 11.40 | 43.19 ± 15.31 | 34.68 ± 10.42 | 286.06 | < 0.001 |
| eHEALS Score | 28.87 ± 2.64 | 20.801 ± 5.02 | 35.67 ± 3.43 | 5272.19 | < 0.001 |

Table S3. Univariate Analysis of the Characteristics of the Participants in Different Latent Classes

of HELIA

| **HELIA Class** | **Class 1** | | **Class 2** | | **Class 3** | | **Class 4** | | **Class 5** | | $\boldsymbol{\chi}^{\boldsymbol{2}}$ | **P** |
| --- | --- | --- | --- | --- | --- | --- | --- | --- | --- | --- | --- | --- |
| **Variable** | **N** | **%** | **N** | **%** | **N** | **%** | **N** | **%** | **N** | **%** | **Test** |  |
| **Gender** | 2635 |  | 1240 |  | 1511 |  | 1330 |  | 640 |  | $\boldsymbol{\chi}^{\boldsymbol{2}}$=37.68 | <0.01 |
| Female | 1915 | 72.7% | 874 | 70.5% | 1094 | 72.4% | 888 | 66.8% | 399 | 62.3% |  |  |
| Male | 720 | 27.3% | 366 | 29.5% | 417 | 27.6% | 442 | 33.2% | 241 | 37.7% |  |  |
| **Educational level** | 2635 |  | 1240 |  | 1511 |  | 1330 |  | 640 |  | $\boldsymbol{\chi}^{\boldsymbol{2}}$=1259.93 | <0.01 |
| Primary school | 88 | 3.3% | 130 | 10.5% | 36 | 2.4% | 147 | 11.1% | 228 | 35.6% |  |  |
| Secondary school | 273 | 10.4% | 256 | 20.6% | 160 | 10.6% | 281 | 21.1% | 162 | 25.3% |  |  |
| Diploma | 819 | 31.1% | 477 | 38.5% | 521 | 34.5% | 495 | 37.2% | 169 | 26.4% |  |  |
| University | 1455 | 55.2% | 377 | 30.4% | 794 | 52.5% | 407 | 30.6% | 81 | 12.7% |  |  |
| **Living location** | 2635 |  | 1240 |  | 1511 |  | 1330 |  | 640 |  | $\boldsymbol{\chi}^{\boldsymbol{2}}$=33.15 | <0.05 |
| City | 2084 | 79.1% | 911 | 73.5% | 1193 | 79% | 982 | 73.8% | 462 | 72.2% |  |  |
| Rural | 551 | 20.9% | 329 | 26.5% | 318 | 21% | 348 | 26.2% | 178 | 27.8% |  |  |
| **Marital status** | 2635 |  | 1240 |  | 1511 |  | 1330 |  | 640 |  | $\boldsymbol{\chi}^{\boldsymbol{2}}$=18.26 | <0.05 |
| Single | 543 | 20.6% | 213 | 17.2% | 308 | 20.4% | 263 | 19.8% | 99 | 15.5% |  |  |
| Married | 2070 | 78.6% | 1011 | 81.5% | 1190 | 78.8% | 1054 | 79.2% | 530 | 82.8% |  |  |
| Divorced | 22 | 0.8% | 16 | 1.3% | 13 | 0.9% | 13 | 1% | 11 | 1.7% |  |  |

Table S4. Univariate Analysis of the Characteristics of the Participants in Different Latent Classes

based on eHEALS data

| eHEALS | Class 1 | | Class 2 | | Class 3 | |  |  |
| --- | --- | --- | --- | --- | --- | --- | --- | --- |
| Variable | **N** | **Percent** | **N** | **Percent** | **N** | **Percent** | **Test** | **P-value** |
| **Gender** | 1930 |  | 1100 |  | 4676 |  | X2=16.52 | <0.001 |
| Female | 1303 | 67.5% | 722 | 65.6% | 3322 | 71% |  |  |
| Male | 627 | 32.5% | 378 | 34.4% | 1354 | 29% |  |  |
| **Educational Level** | 1930 |  | 1100 |  | 4676 |  | X2=483.16 | <0.001 |
| Primary School | 211 | 10.9% | 244 | 22.2% | 242 | 5.2% |  |  |
| Secondary School | 337 | 17.5% | 246 | 22.4% | 628 | 13.4% |  |  |
| Diploma | 665 | 34.5% | 355 | 32.3% | 1591 | 34% |  |  |
| University | 717 | 37.2% | 255 | 23.2% | 2215 | 47.4% |  |  |
| **Living Location** | 1930 |  | 1100 |  | 4676 |  | X^2^=25.17 | <0.001 |
| City | 1437 | 74.5% | 792 | 72% | 3661 | 78.3% |  |  |
| Rural | 493 | 25.5% | 308 | 28% | 1015 | 21.7% |  |  |
| **Marital Status** | 1930 |  | 1100 |  | 4676 |  | X2=1.82 | 0.77 |
| Single | 375 | 19.4% | 211 | 19.2% | 930 | 19.9% |  |  |
| Married | 1537 | 79.6% | 875 | 79.5% | 3705 | 79.2% |  |  |
| Divorced | 18 | 0.9% | 14 | 1.3% | 41 | 0.9% |  |  |
